# A Systematic Review of the Level of Evidence in Economic Evaluations of Medical Devices: The Example of Vertebroplasty and Kyphoplasty

**DOI:** 10.1371/journal.pone.0144892

**Published:** 2015-12-10

**Authors:** Nicolas Martelli, Capucine Devaux, Hélène van den Brink, Judith Pineau, Patrice Prognon, Isabelle Borget

**Affiliations:** 1 Pharmacy Department, Georges Pompidou European Hospital, AP-HP, 20 rue Leblanc, 75015, Paris, France; 2 GRADES, Université Paris-Sud, Université Paris-Saclay, 5 rue Jean-Baptiste Clément, 92290, Châtenay-Malabry, France; 3 Department of Health Economics, Gustave Roussy Institute, 114, rue Edouard-Vaillant, 94805, Villejuif, France; University of Milan, ITALY

## Abstract

**Context:**

Economic evaluations are far less frequently reported for medical devices than for drugs. In addition, little is known about the quality of existing economic evaluations, particularly for innovative devices, such as those used in vertebroplasty and kyphoplasty.

**Objective:**

To assess the level of evidence provided by the available economic evaluations for vertebroplasty and kyphoplasty.

**Data Sources:**

A systematic review of articles in English or French listed in the MEDLINE, PASCAL, COCHRANE and National Health Service Economic Evaluation databases, with limits on publication date (up to the date of the review, March 2014).

**Study Selection:**

We included only economic evaluations of vertebroplasty or kyphoplasty. Editorial and methodological articles were excluded.

**Data Extraction:**

Data were extracted from articles by two authors working independently and using two analysis grids to measure the quality of economic evaluations.

**Data Synthesis:**

Twenty-one studies met our inclusion criteria. All were published between 2008 and 2014. Eighteen (86%) were full economic evaluations. Cost-effectiveness analysis (CEA) was the most frequent type of economic evaluation, and was present in 11 (52%) studies. Only three CEAs complied fully with the British Medical Journal checklist. The quality of the data sources used in the 21 studies was high, but the CEAs conforming to methodological guidelines did not use high-quality data sources for all components of the analysis.

**Conclusions:**

This systematic review shows that the level of evidence in economic evaluations of vertebroplasty and kyphoplasty is low, despite the recent publication of a large number of studies. This finding highlights the challenges to be faced to improve the quality of economic evaluations of medical devices.

## Introduction

Health technology assessment (HTA) is increasingly used to support healthcare allocation decisions in most health systems [1]. HTA ensures that decisions are based on the best evidence available. Indeed, health technologies are frequently seen as an important driver of rising health expenditure and one of the purposes of HTA is to analyze the economic implications of adopting new technologies [2]. Economic evaluations are thus an important source of information about health technologies. However, the methods currently used for economic evaluations of health products were initially developed with drugs in mind and are often unsuitable for medical devices [3]. Economic evaluations involve the collection of cost data, the establishment of clinical effectiveness and sensitivity analyses, but several of the specific features of medical devices may complicate such analyses [4]. First, devices evolve more rapidly than drugs. Consequently, new products are regularly released onto the market, with probable effects on prices and major effects on cost evaluation [5,6]. In addition, medical devices have wider economic implications that must be assessed, such as an impact on organization or a need for training. It is also challenging to design clinical trials for medical devices, and assessment methods must be modified for such trials [7]. It is often difficult to recruit large samples of patients, because most devices are suitable for use in only a limited number of patients. The procedures currently used to minimize study bias for drugs, such as blinding and randomization, are not always possible for surgical devices, for ethical or practical reasons [8]. Some devices are implantable and require very long-term follow-up that may be difficult to implement. Finally, device-operator interactions are very strong and can have a significant impact on the extent to which clinical results can be generalized. For all these reasons, large randomized controlled trials (RCTs) providing valid and unbiased estimates of efficacy are difficult to achieve for medical devices.

As a result, far fewer economic evaluations of medical devices than of drugs have been published [2,9]. In view of the constraints associated with medical devices, questions also remain about the level of evidence provided by the few economic evaluations available. It would also be useful to focus on surgical procedures in which innovative devices are used and for which economic evaluations through a HTA assessment process are clearly valuable. We identified vertebroplasty and kyphoplasty as particularly good case studies in this context. Both are procedures for treating the consequences of osteoporosis, a disease that is becoming increasing frequent with the aging of the population in Western countries [10]. Indeed, osteoporosis is the most common cause of vertebral compression fractures (VCFs), which have short-term effects, such as acute and chronic pain, but are also associated with long-term morbidity [11]. The management of VCFs is therefore a matter of public health concern. Vertebroplasty and kyphoplasty are minimally invasive procedures, with broad economic implications, due to their impact on the organization of healthcare institutions, the need to train medical teams and the ability of these techniques to decrease the frequency of postoperative complications. They do not treat the primary disease itself, but can improve the quality of life of patients. Finally, the cost of the medical devices used for these procedures is higher than that of the standard treatment for VCF pain (i.e. optimal pain management (OPM) with oral analgesics).

The aim of this systematic review was to assess the level of evidence provided by the available economic evaluations for vertebroplasty and kyphoplasty. In this work, we did not aim to assess the cost-effectiveness or value of these procedures. Our aim was purely to illustrate the level of published evidence available for economic evaluations of medical devices through a particular example.

## Materials and Methods

### Study Selection

A systematic literature review was conducted to identify economic evaluations of vertebroplasty and kyphoplasty. Relevant studies were identified from the following databases: MEDLINE, PASCAL, COCHRANE and the National Health Service Economic Evaluation Database (NHS EED) at the University of York. Searches were undertaken in March 2014, with no limitations concerning publication date. The search strategy was developed in MEDLINE and was then adapted for the other databases. The search terms employed included: Cementoplasty/economics[Mesh]; "Costs and Cost Analysis"[Mesh] AND "Cementoplasty"[Mesh]; "Economics"[Mesh] AND "Cementoplasty"[Mesh]; cost[Title/Abstract] AND kyphoplasty[Title/Abstract]; cost[Title/Abstract]) AND vertebroplasty[Title/Abstract]. We are aware that “cementoplasty” is a broad term encompassing vertebroplasty and kyphoplasty, but also other procedures, such as osteoplasty and sacroplasty.

We followed the Preferred Reporting Items for Systematic Reviews and Meta-Analyses (PRISMA) guidelines for this systematic review (S1 Checklist) [12]. The titles and abstracts were first screened independently by CD and NM, to determine whether they met the inclusion or exclusion criteria. We thus identified and excluded duplicate and irrelevant abstracts. We then carried out a full-text review of all the texts retained at the end of the initial screening step. Studies were included if they met the following criteria: (i) the study conducted was an economic evaluation on vertebroplasty or kyphoplasty, (ii) the article concerned was written in English or French. We excluded editorial or methodological studies and articles for which no full-text format was available.

### Data Collation and Analysis

We used two different methods to evaluate the level of evidence provided by the studies included. This methodology was inspired by previous works on the quality of economic evaluations in healthcare [13].

First, we considered the extent to which the study complied with methodological recommendations from internationally recognized guidelines published by Drummond *et al*. and generally referred to as the British Medical Journal (BMJ) checklist [14,15]. These recommendations provide general guidance about the way in which the results of economic evaluations should be reported. The authors are required to indicate the study perspective (the point of view from which the study is conducted), to provide a description of comparators, to specify the type of costs used for the analysis, to report the incremental cost-effectiveness ratio (ICER), to perform uncertainty analysis and to disclose funding sources. Using this method, two researchers (CD and NM) extracted the following data independently from each of the studies included: authors' names, publication year, country of origin, type of economic evaluation, perspective, time window of the study, description of comparators, type of costs used, ICER calculation, uncertainty analysis and source of funding. The different types of economic evaluation carried out were classified as follows: (i) partial economic evaluation (PEE), (ii) cost-minimization analysis (CMA), (iii) cost-effectiveness analysis (CEA), (iv) cost-utility analysis (CUA) and (v) cost-benefit analysis (CBA) [14]. The studies were considered to include a PEE if the comparison did not include both the costs and consequences of therapeutic strategies [14]. By contrast, CMA, CEA, CUA and CBA were considered to be full economic evaluations. In cases of discordant classifications, the two researchers discussed discrepancies until a consensus was reached.

We then used the hierarchy scale developed by Cooper *et al*.[16] to assess the quality of evidence from the data sources used in the studies included. With this scale, study quality is gauged from the model used to obtain the data. The hierarchy scale is presented in S1 Table. For example, the data used to assess patient outcomes (clinical effect and safety) are awarded a score of one to six where “one” corresponds to the highest quality study design (meta-analysis of RCTs with direct comparison between comparator therapies) and “six” is the lowest quality study design (expert opinion). A score of nine is attributed if the data sources are not clearly indicated. Scores were again attributed by two researchers (CD and NM) working independently and discrepancies were discussed until a consensus was reached. Finally, we grouped the ranks into three quality levels: A, B and C. Level A corresponds to the highest level of evidence quality, covering ranks 1 and 2. Level B corresponds to an intermediate level of evidence quality, covering ranks 3 and 4. Ranks 5, 6 and 9 were grouped together in level C, corresponding to the lowest level of evidence quality.

## Results

### Study Selection

**Fig 1:**
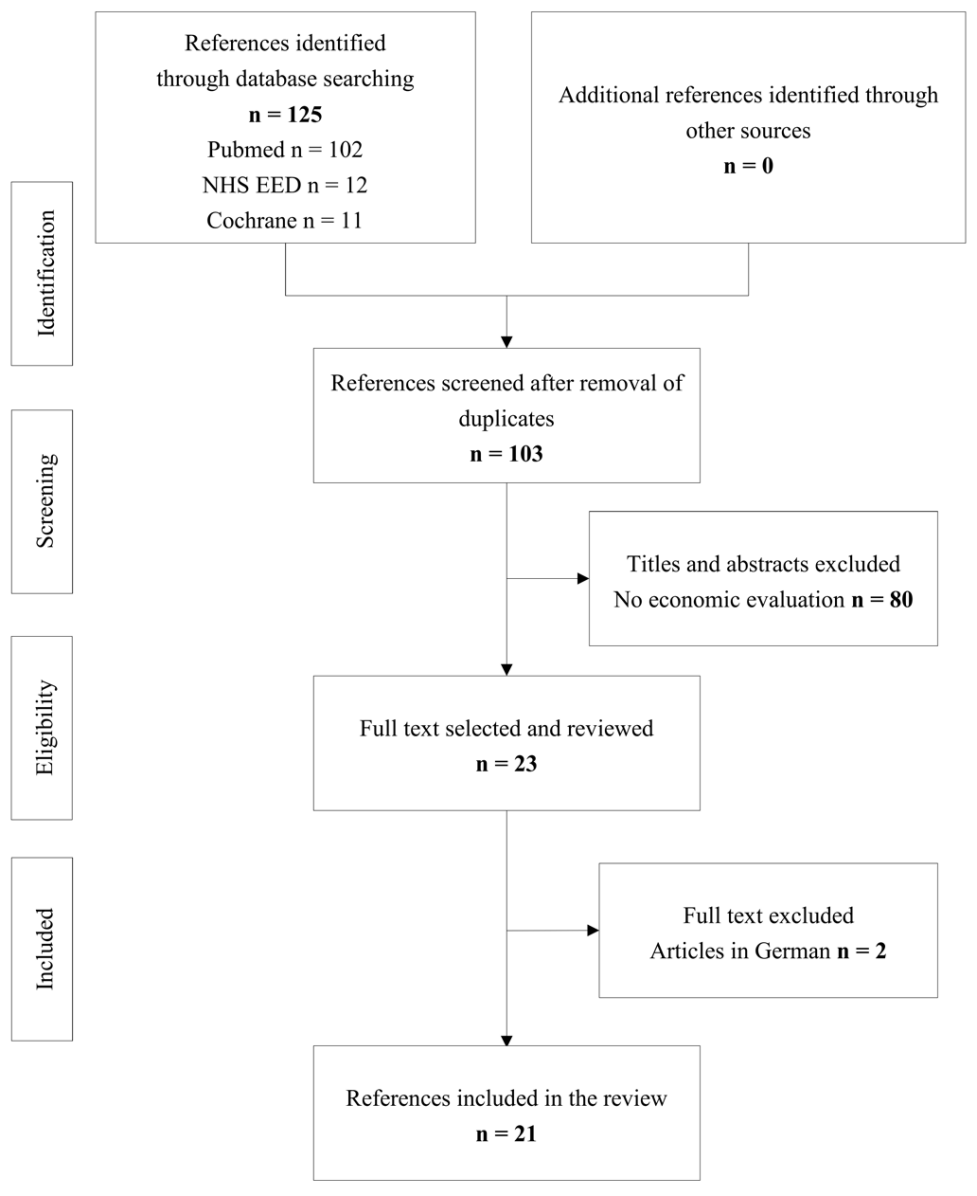
PRISMA flow chart for study inclusion.

In total, we identified 103 unique references. An initial review of titles and abstracts led to the exclusion of 80 references, in which no economic evaluation was conducted. We therefore carried out full-text reviews of 23 articles, two of which were excluded because they were published in German (Fig 1). All 21 references retained were published between 2008 and 2014, with a peak in the number of relevant articles published in 2013 (7/21, 33%).

### Characteristics of the Studies Included

The characteristics of the 21 economic evaluations are summarized in Table 1 [17–37]. Eleven of the 21 studies included (52%) contained CEAs, five (24%) contained CMAs, three (14%) contained PEEs and two (10%) contained CUAs. Thus, 18 (86%) of the studies included contained full economic evaluations.

**Table 1:** Characteristics of the 21 economic evaluations reviewed.

Reference	Year	Country	Economic evaluation	Perspective	Cost included	Comparator	Uncertainty analysis	ICER	Sources of funding	Time window (months)
**Becker *et al*. [** 17 **]**	2011	Austria	CEA	Hospital	Direct	Yes	No	No	Private	48
**Chew *et al*. [** 18 **]**	2013	UK	CUA	Societal	Direct	No	No	Yes	None	4
**Edidin *et al*. [** 19 **]**	2012	USA	CEA	Societal	Total	Yes	Yes	Yes	Private	48
**Flug *et al*. [** 20 **]**	2013	USA	PEE	Hospital	Total	Yes	No	N/A	None	30
**Fritzell *et al*. [** 21 **]**	2011	Sweden	CEA	Societal	Total	Yes	Yes	Yes	Private	35
**Gan *et al*. [** 22 **]**	2013	China	PEE	Hospital	Direct	Yes	No	N/A	Public	84
**Goz *et al*. [** 23 **]**	2013	USA	CEA	Societal	Direct	Yes	No	No	None	72
**Gray *et al*. [** 24 **]**	2008	USA	PEE	Societal	Direct	No	No	N/A	Public	48
**Hart *et al*.[** 25 **]**	2008	USA	CMA	Hospital	Direct	Yes	Yes	N/A	None	25
**Itagaki *et al*. [** 26 **]**	2012	USA	CEA	Societal	Total	Yes	No	N/A	Public	120
**Lad *et al*.[** 27 **]**	2009	USA	CMA	Hospital	Direct	Yes	No	N/A	None	132
**Lange *et al*. [** 28 **]**	2014	Germany	CEA	Societal	Direct	Yes	No	N/A	Private	60
**Masala *et al*. [** 29 **]**	2008	Italy	CEA	Hospital	Direct	Yes	No	Yes	None	12
**Mehio *et al*. [** 30 **]**	2011	USA	CMA	Hospital	Total	Yes	No	N/A	Private	24
**Ong *et al*.[** 31 **]**	2013	USA	CMA	Societal	Direct	Yes	No	N/A	Private	60
**Ström *et al*. [** 32 **]**	2010	Sweden	CEA	Societal	Total	Yes	Yes	Yes	Private	12
**Svedbom *et al*. [** 33 **]**	2012	Sweden	CEA	Societal	Direct	Yes	Yes	Yes	Private	24
**Takura *et al*. [** 34 **]**	2013	Japan	CUA	Societal	Direct	No	Yes	Yes	None	12
**Tang *et al*. [** 35 **]**	2011	China	CEA	Hospital	Direct	Yes	No	No	None	46
**Voidey *et al*. [** 36 **]**	2013	France	CMA	Societal	Direct	No	No	N/A	None	32
**Zampini *et al*. [** 37 **]**	2010	USA	CEA	Hospital	Direct	Yes	No	No	None	12

CEA: Cost-effectiveness analysis; CMA: Cost-minimization analysis; CUA: Cost-utility analysis; ICER: Incremental cost-effectiveness ratio; N/A: Not applicable; PEE: Partial economic evaluation

Information about compliance with the BMJ checklist for the reporting of economic evaluations is presented in Table 2. Two different perspectives were found in the studies included: societal and hospital perspectives. The majority of the studies considered (57%) carried out economic analyses from a societal perspective. Four of the 21 studies (19%) provided no comparators. Most studies (71%) used direct costs for the analysis. The time windows considered ranged from 4 months to 11 years, and the median study duration was 35 months. The study with the longest time window was classified as a CMA. Four CEAs and one CUA presented an ICER calculation together with an uncertainty analysis. Only three CEAs carried out the analysis from a societal perspective, used a comparator and considered total costs. One of these three studies concluded that kyphoplasty was cost-effective, relative to vertebroplasty, for the treatment of VCFs [19]. Another established that, for patients with an acute vertebral fracture due to osteoporosis, it was not possible to demonstrate the cost-effectiveness of kyphoplasty relative to standard medical treatment [21]. Finally, the third study concluded that kyphoplasty for the treatment of painful hospitalized vertebral fractures was cost-effective relative to non-surgical management [32]. However, it should be borne in mind that these conclusions are valid solely in the context in which these studies were carried out.

**Table 2:** Compliance of the studies reviewed with international recommendations for reporting economic evaluations.

Recommendation	Number of studies in which the recommendation was followed (%)				
	Total [*n* (%)]	CEA [*n* (%)]	CMA [*n* (%)]	CUA [*n* (%)]	PEE [*n* (%)]
Perspective specified	21/21 (100%)	11/11 (100%)	5/5 (100%)	2/2 (100%)	3/3 (100%)
*Societal perspective*	12/21 (57%)	7/11 (64%)	2/5 (40%)	2/2 (100%)	1/3 (33%)
*Hospital perspective*	9/21 (43%)	4/11 (36%)	3/5 (60%)		2/3 (66%)
Description of comparators	17/21 (81%)	11/11 (100%)	4/5 (80%)		2/3 (66%)
Type of costs used specified	21/21 (100%)	11/11 (100%)	5/5 (100%)	2/2 (100%)	3/3 (100%)
*Direct costs*	15/21 (71%)	7/11 (64%)	4/5 (80%)	2/2 (100%)	2/3 (66%)
*Total costs*	6/21 (29%)	4/11 (36%)	1/5 (20%)		1/3 (33%)
ICER calculation	7/11 (64%)	5/9 (56%)		2/2 (100%)	
Uncertainty analysis performed	6/21 (29%)	4/11 (36%)	1/5 (20%)	1/2 (50%)	
Sources of funding specified	21/21 (100%)	11/11 (100%)	5/5 (100%)	2/2 (100%)	3/3 (100%)
*No funding source*	10/21 (48%)	4/11 (36%)	3/5 (60%)	2/2 (100%)	1/3 (33%)
*Private funding*	8/21 (38%)	6/11 (55%)	2/5 (40%)		
*Public funding*	3/21 (14%)	1/11 (9%)			2/3 (66%)

CEA: cost-effectiveness analysis; CMA: cost-minimization analysis; CUA: Cost-utility analysis; ICER: Incremental cost-effectiveness ratio; PEE: Partial economic evaluation.

Eight of the 12 studies with a societal perspective (67%) used direct costs for the analysis. Funding sources were specified in all studies. There was no direct funding for 10 (48%) studies. Only three (14%) studies were funded by the public sector. The eight (38%) remaining studies were funded by profit-making organizations (medical device companies in all cases).

### Quality of the Data Sources Used in the Economic Evaluations Reviewed

We used the hierarchy scale of Cooper *et al*. to classify the data sources used in the 21 studies included. The details of this analysis are shown in Table 3. The data sources for determining clinical effect sizes and safety were of the highest level of quality (A) for 12 studies (57%), whereas one study (5%) used data sources of poor quality (C). For the baseline clinical data, nine studies (43%) used poor-quality data (level C). Six of these nine studies did not indicate their sources. Only one (5%) study specifically analyzed administrative databases covering patients from the jurisdiction of interest. The data sources used to analyze resource use were of high quality for almost all studies, as 19 studies (91%) used level A information sources. The data sources for determining costs were also generally of high quality, with 15 studies (71%) using information sources of ranks 1 and 2. Nevertheless, the data used in two studies (10%) were extracted from level C data sources, and one of these two studies did not clearly state the sources of the information used.

**Table 3:** Quality of evidence used in the 21 economic evaluations reviewed.

**Quality of evidence**	**Hierarchy of evidence**	Clinical effect sizes/adverse events and complications [*n* (%)]	Baseline clinical data [*n* (%)]	Resource use [*n* (%)]	Costs [*n* (%)]	Utilities [*n* (%)]
**A**	1+	4 (19%)				
	1	5 (24%)	1 (5%)	2 (10%)	2 (10%)	3 (43%)
	2	3 (14%)	5 (24%)	17 (81%)	13 (62%)	
**B**	3		3 (14%)	1 (5%)	1 (5%)	3 (43%)
	4	8 (38%)	3 (14%)	1 (5%)	3 (14%)	
**C**	5				1 (5%)	1 (14%)
	6	1 (5%)	3 (14%)			
	9		6 (29%)		1 (5%)	

A: highest level of evidence quality; B: intermediate level of evidence quality; C: lowest level of evidence quality

By combining the two analyses, we focused on the quality of evidence used by the three CEAs in complete accordance with methodological recommendations. One study used sources from level A for each data component except for the determination of baseline clinical data, but this was not clearly stated in the paper. Another study used level A sources for all data components except clinical effect size and safety, for which the data were extracted from sources with a rank of 4 (level B). Finally, the data for the third study were extracted from level A sources for two components and from level B sources for three components.

## Discussion

This systematic review identified 21 economic evaluations of vertebroplasty and kyphoplasty. We found that a wide variety of methods had been used for economic evaluations and their reporting, with major differences between studies in the type of economic evaluation, perspective used, costs, time horizons and quality of evidence used to perform the analysis.

Most of the studies did not fully comply with the BMJ checklist. One of the most common flaws observed was a lack of ICER calculation. ICER is particularly useful for decision-makers, because it simplifies the comparison of two mutually exclusive interventions and clearly indicates the additional benefits to be gained from an intervention [14]. If ICER is to be a valuable tool for decision-making support, it must be followed by a sensitivity analysis to assess the uncertainty on the calculation and to determine the robustness of the conclusions drawn. Again, very few of the CEAs included proposed a sensitivity analysis, and this rendered the results difficult to interpret. It is difficult to determine whether ICER and/or sensitivity analyses were not provided due to missing data or due to an inability of the authors to perform such analyses correctly. The rationale behind the decision not perform such evaluations was only very rarely explained. Total costs were thus analyzed from a societal perspective in only a minority of studies. Such evaluations should, as far as possible, be carried out from a societal perspective when assessing the overall costs of the interventions. In the case of vertebroplasty and kyphoplasty, the use of a societal perspective is particularly relevant because productivity losses are a major component of the impact to be assessed [38]. The use of such a perspective would ensure that indirect costs associated with these interventions were not dismissed out of hand. Finally, all the studies included clearly specified the source of funding. This is a positive point, but it also raises ethical questions, because most of the studies complying with the BMJ checklist were funded directly by medical device companies. Several authors have already suggested that studies sponsored by industry are much more likely to reach favorable qualitative conclusions than similar studies funded by not-for-profit organizations [39–41]. There may be a number of reasons for this, such as the submission only of analyses with positive results or the selection of study strategies that industry is likely to fund. Unfortunately, the level of involvement of the sponsor in the study was not clearly indicated in any of the studies included. For example, it would have been useful to know whether the final version of the manuscript had to be approved by the sponsor before its submission [40].

The quality of the data sources used for the economic evaluations was generally high. However, this observation might conceal significant differences between data components. For example, information on resource use was determined with high-quality data in almost all studies, whereas the sources of baseline clinical data were frequently of poor quality or not stated. One possible explanation for these discrepancies is that some data are undoubtedly harder to obtain than others, particularly for medical devices [42]. For example, it is difficult to account for all the cost items associated with medical device use, because these products often have far-reaching consequences for organization affecting many components [4]. Caution is also required concerning the implementation of the hierarchy scale applied here. Data from level A sources may well be of better quality than those from lower level sources. However, there is no guarantee that they were generated without bias. For example, two RCTs, FREE and VERTOS, were widely used in the 21 economic evaluations for the determination of clinical effect sizes and safety [43,44]. In a recent HTA report from the National Institute for Health Research, nine RCTs on percutaneous vertebroplasty and kyphoplasty, including FREE and VERTOS [45], were reviewed in depth. The authors identified several methodological issues and biases in both these studies, including a lack of blinding and attrition bias. We have already mentioned the many difficulties involved in RCTs on medical devices, including device-operator interactions, small sample sizes and the need for long-term follow-up [8]. Caution is therefore required when using RCTs on medical devices as data sources, because RCTs may not necessarily be based on unbiased data. We also found that even the CEAs complying fully with the BMJ checklist did not use high-quality sources of data for all the necessary components of the analysis. Thus, none of the studies reviewed here carried out a full economic evaluation reported complete information and used high-quality data for all analysis components. Other studies have identified methodological flaws in economic evaluations in healthcare, regardless of the type of intervention or health product [46–49]. It is, therefore, difficult to attribute these flaws exclusively to the type of health product studied here: medical devices. The methodological quality of economic evaluations is a global issue and the particular features of medical devices are not sufficient to account for the use of poor-quality sources revealed here.

Finally, this review has several limitations worthy of discussion. First, we restricted the selection of studies to those written in French and English. This led to the elimination of two studies written in German that were potentially relevant for our analysis. We used several databases, but some economic evaluation studies are probably published in data sources other than scientific journals. In this review, we focused exclusively on articles published in scientific journals and did not include economic evaluations from gray literature. Finally, assessment of the quality of economic evaluations is difficult and can be subjective. This point has already been highlighted by a number of authors [50,51].

## Conclusion

Our findings show that the level of evidence used in economic evaluations of vertebroplasty/kyphoplasty is quite low despite the large number of studies published recently. We believe that this work is a good proxy, reflecting the quality of economic evaluations available for innovative medical devices. However, further studies on other medical devices are required to confirm these results. Finally, if efforts are not made to improve the quality of economic evaluations of medical devices and their reporting, then the resource allocation decisions based on these evaluations will remain uncertain.

## Supporting Information

S1 Checklist PRISMA statement. (DOC)

S1 Dataset BMJ checklist completed. (XLS)

S2 Dataset Cooper checklist completed. (XLS)

S1 Table Hierarchy scale for data sources in economic evaluations from Cooper et al. (DOCX)
