# Supplementary material for: A Systematic Review of the Level of Evidence in Economic Evaluations of Medical Devices: The Example of Vertebroplasty and Kyphoplasty
Source: PLoS One. 2015 Dec 10;10(12):e0144892. doi: 10.1371/journal.pone.0144892 (PMC4675526; doi:10.1371/journal.pone.0144892)
Supplement: S1 Table — (DOCX) [file pone.0144892.s004.docx]

**Table 1.** Hierarchy scale for data sources in economic evaluations from Cooper *et al.* [16]

| **Clinical effect sizes/adverse events and complications** | |
| --- | --- |
| **1+** | Meta-analysis of RCTs with direct comparison between comparator therapies, measuring final outcomes |
| **1** | Single RCT with direct comparison between comparator therapies, measuring final outcomes |
| **2+** | Meta-analysis of RCTs with direct comparison between comparator therapies, measuring surrogate outcomes |
|  | Meta-analysis of placebo-controlled RCTs with similar trial populations, measuring the final outcomes for each individual therapy |
| **2** | Single RCT with direct comparison between comparator therapies, measuring the surrogate outcomes |
|  | Single placebo-controlled RCTs with similar trial populations, measuring the final outcomes for each individual therapy |
| **3+** | Meta-analysis of placebo-controlled RCTs with similar trial populations, measuring the surrogate outcomes |
| **3** | Single placebo-controlled RCTs with similar trial populations, measuring the surrogate outcomes for each individual therapy |
| **4** | Case control or cohort studies |
| **5** | Non-analytic studies (e.g. case reports, case series) |
| **6** | Expert opinion |
| **9** | Not clearly stated |
| **Baseline clinical data (if applicable)** | |
| **1** | Case series or analysis of reliable administrative databases specifically conducted for the study covering patients solely from the jurisdiction of interest |
| **2** | Recent case series or analysis of reliable administrative databases covering patients solely from the jurisdiction of interest |
| **3** | Recent case series or analysis of reliable administrative databases covering patients solely from another jurisdiction |
| **4** | Old case series or analysis of reliable administrative databases. Estimates from RCTs. |
| **5** | Estimates from previously published economic analyses: unsourced |
| **6** | Expert opinion |
| **9** | Not clearly stated |
| **Resource use** | |
| **1** | Prospective data collection or analysis of reliable administrative data for specific study |
| **2** | Recently published results of prospective data collection or recent analysis of reliable administrative data: same jurisdiction |
| **3** | Unsourced data from previous economic evaluations: same jurisdiction |
| **4** | Recently published results of prospective data collection or recent analysis of reliable administrative data: different jurisdiction |
| **5** | Data source not known: different jurisdiction |
| **6** | Expert opinion |
| **9** | Not clearly stated |
| **Costs** | |
| **1** | Cost calculations based on reliable databases or data sources conducted for specific study: same jurisdiction |
| **2** | Recently published cost calculations based on reliable databases or data course: same jurisdiction |
| **3** | Data source not known: same jurisdiction |
| **4** | Using charge (price) rather than cost when societal perspective was adopted |
| **5** | Recently published cost calculations based on reliable databases or data sources: different jurisdiction |
| **6** | Data source not known: different jurisdiction |
| **9** | Not clearly stated |
| **Utilities (if applicable)** | |
| **1** | Direct utility assessment for the specific study from a sample either: |
|  | (a) of the general population, or |
|  | (b) with knowledge of the disease(s) of interest, or |
|  | (c) of patients with the disease(s) of interest |
|  | Indirect utility assessment from specific study from patient sample with disease(s) of interest, using a tool validated for the patient population |
| **2** | Indirect utility assessment from a patient sample with disease(s) of interest, using a tool not validated for the patient population |
| **3** | Direct utility assessment from a previous study from a sample either: |
|  | (a) of the general population, or |
|  | (b) with knowledge of the disease(s) of interest, or |
|  | (c) of patients with the disease(s) of interest |
|  | Indirect utility assessment from previous study from patient sample with disease(s) of interest, using a tool validated for the patient population |
| **4** | Data source not known: method of elicitation unknown |
| **5** | Patient preference values obtained from a visual analogue scale |
| **6** | Delphi panels, expert opinion |
| **9** | Not clearly stated |
